# A Study on the Femoral Neck-Shaft Angle in an Adult Sample From Andhra Pradesh: Osteological and Radiological Measurements

**DOI:** 10.7759/cureus.82100

**Published:** 2025-04-11

**Authors:** D Ranzeetha, Pratyusha Challa, Vasanthi Lakkireddy, K V Pavana Kumari, K Lakshmi Kumari, D Madhavi

**Affiliations:** 1 Anatomy, Guntur Medical College, Guntur, IND; 2 Anatomy, Government Medical College, Ongole, IND; 3 Anatomy, Government Medical College, Rajamahendravaram, IND; 4 Anatomy, Government Medical College, Visakhapatnam, IND

**Keywords:** femoral anatomy, femoral neck-shaft angle, hip biomechanics, osteological measurement, radiological measurement

## Abstract

The femoral neck-shaft angle (NSA) is a crucial anatomical parameter that plays a significant role in hip biomechanics, lower limb alignment, and load distribution across the hip joint. Variations in femoral NSA are linked to orthopedic conditions and have implications for surgical planning, prosthetic design, and forensic analysis. This study aimed to assess the femoral NSA in an adult sample from Andhra Pradesh using both osteological and radiological methods and to evaluate its variations based on side, gender, and age.

An observational study was conducted from September to November 2024 at Guntur Medical College in Guntur and Government Medical College in Ongole in Andhra Pradesh, India. The study was conducted using osteological measurements from 113 dry femora (54 left, 59 right) and radiological measurements from 30 standardized anteroposterior digital X-rays of adult subjects aged between 20 and 80 years. Femoral NSA was measured bilaterally using a goniometer and image analysis software. Age- and gender-wise analyses were not possible for osteological measurements. Data were analyzed using IBM SPSS Statistics software, version 26 (IBM Corp., Armonk, NY). Independent t-tests assessed side- and gender-based differences, while one-way ANOVA evaluated age-related variation. Pearson’s correlation was employed to compare measurement methods, with a p-value of less than 0.05 considered statistically significant.

In osteological assessment, the mean NSA was 116.54° ± 7.81° on the left and 119.93° ± 3.85° on the right, with a statistically significant difference (p = 0.0047). In radiological measurements, the NSA was 141.15° ± 6.97° (left) and 137.50° ± 5.72° (right), also significant (p = 0.0307). In the radiological measurement, gender-based analysis showed a significant difference in females (p = 0.0385) but not males (p = 0.376). Age-wise, significant right-to-left NSA differences were observed across all age groups, which were 20 to 40 years (p = 0.039), 41 to 60 years (p = 0.048), and 61 to 80 years (p = 0.041). A positive correlation was observed between the two methods (r = 1.0), indicating measurement consistency.

The study establishes normative femoral NSA values for the adult sample from Andhra Pradesh and highlights significant side-specific, gender-based, and age-related variations. The positive correlation between both methods (limited to bilateral variations) supports their reliability in clinical and anatomical assessments. These findings may offer crucial primary reference data for orthopedic procedures, prosthetic alignment, and regional forensic profiling. Future studies with larger, demographically stratified samples and clinical variables are recommended to refine and validate these observations.

## Introduction

The femoral neck-shaft angle (NSA) is a critical anatomical parameter influencing hip biomechanics, weight transmission, and lower limb alignment [1]. It is defined as the angle formed between the femoral neck and the shaft of the femur and plays a significant role in gait mechanics, joint stability, and load distribution across the hip joint [2]. Variations in the femoral NSA have been associated with conditions such as hip osteoarthritis, femoroacetabular impingement, and developmental dysplasia of the hip [3, 4]. Understanding these variations is essential for orthopedic planning, prosthetic design, forensic investigations, and anthropometric studies [5].

The femoral NSA is influenced by multiple factors, including genetics, age, gender, ethnicity, and environmental influences such as physical activity levels and specific professional activities [6]. Additionally, the presence or absence of a third trochanter, an accessory tubercle of the femur, may influence the femoral NSA [7]. Previous studies have reported population-based differences in femoral NSA values, highlighting the need for region-specific reference values [8]. In the Indian population, variations in femoral NSA have been noted compared to Western and African populations, indicating potential implications for surgical interventions, such as hip replacement and fracture fixation [9].

Traditionally, femoral NSA is measured using osteological (dry bone) and radiological (digital X-ray) methods [10]. While osteological measurements provide direct anatomical reference points, radiological assessments are widely used in clinical settings [11]. However, discrepancies may arise due to soft tissue interference, radiographic positioning, and methodological differences. A comparative evaluation of these two methods can enhance our understanding of femoral NSA variations and establish reliable baseline data for medical applications.

This study aims to assess the femoral NSA in an adult sample from the population of Andhra Pradesh using both osteological and radiological measurements. The specific objectives include determining the femoral NSA in dry femur bones and assessing side-specific variations, evaluating femoral NSA using digital radiographs, analyzing variations based on age and gender, and comparing osteological and radiological measurements to identify potential differences.

## Materials and methods

Study design and setting

This prospective observational study was conducted at the Department of Anatomy, Guntur Medical College, Guntur, and Government Medical College, Ongole, in Andhra Pradesh, India, over three months (September 2024 to November 2024). The study involved both osteological and radiological assessments to determine the femoral NSA in the adult population of Andhra Pradesh.

Sample size and selection criteria

Osteological Data (Dry Femur Bones)

One hundred and thirteen dry femur bones (54 left and 59 right) were obtained from the Department of Anatomy at both institutions for osteological analysis. The bones included in the study were intact, with no structural defects at the proximal end. Femora exhibiting fractures, deformities, or missing proximal ends were excluded to ensure measurement accuracy. However, the age, gender, and medical/surgical history of the dry femur bones were not available.

Radiological Data (Digital X-ray Images)

For radiological analysis, 30 radiographic images of male and female subjects aged 20 to 80 years were collected from the Department of Radiology at both Guntur Medical College, Guntur, and Government Medical College, Ongole. The inclusion criteria for radiographs required standardized anteroposterior (AP) images of the proximal femur from subjects with no history of femoral fractures, hip deformities, or avascular necrosis. Radiographs showing fractures, congenital anomalies, or pathological conditions affecting the femur were excluded from the study.

Measurement techniques

Osteological Measurement

**Figure 1:**
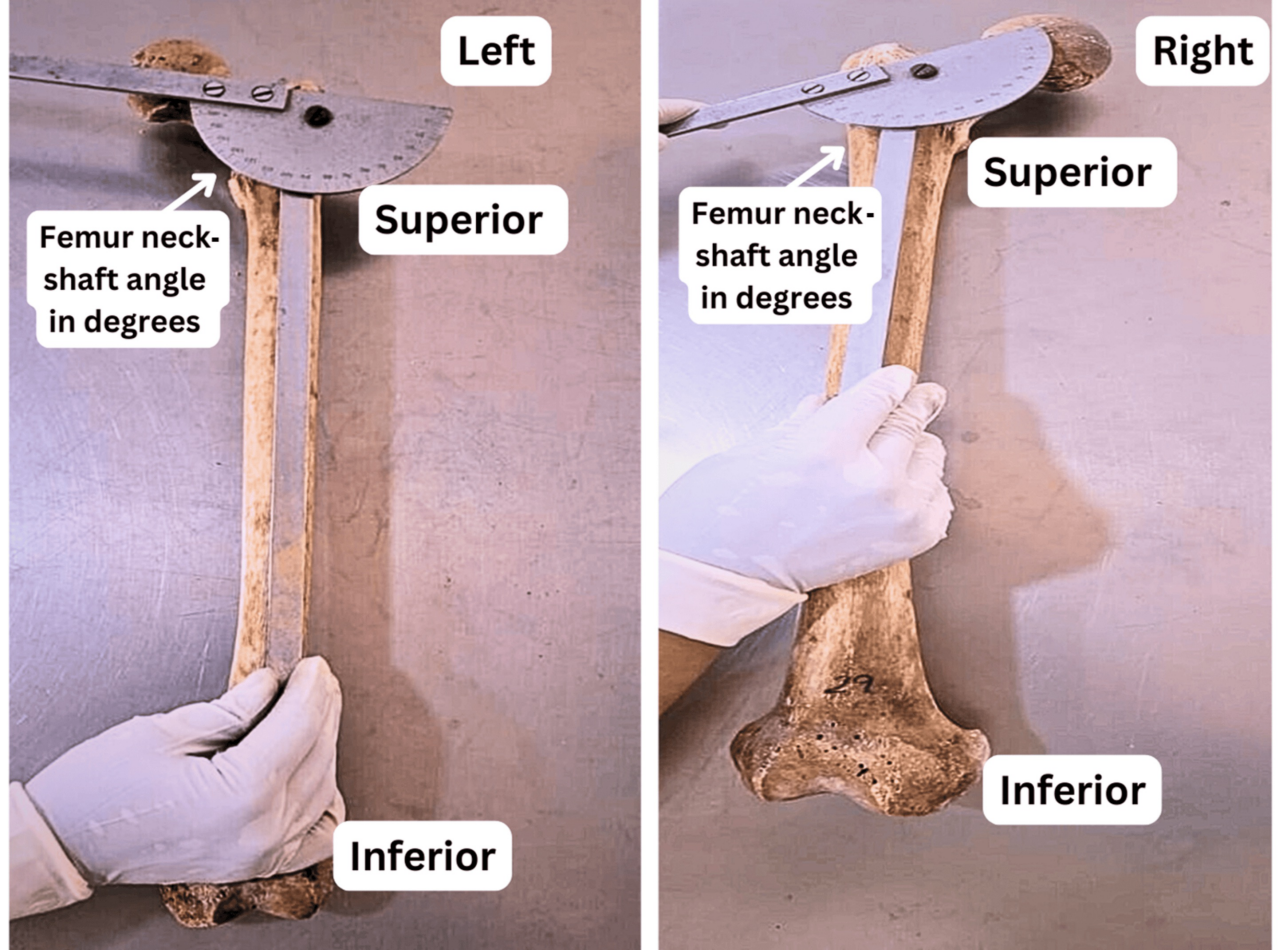
Left and right femurs showing measurements of the femoral neck-shaft angle

The femoral NSA was measured directly on dry femur bones using a goniometer (Figure 1). To minimize measurement errors, independent observers took each reading twice, and the average of the two readings was recorded for analysis. The angle was measured to the nearest degree to ensure precision.

Radiological Measurement

**Figure 2:**
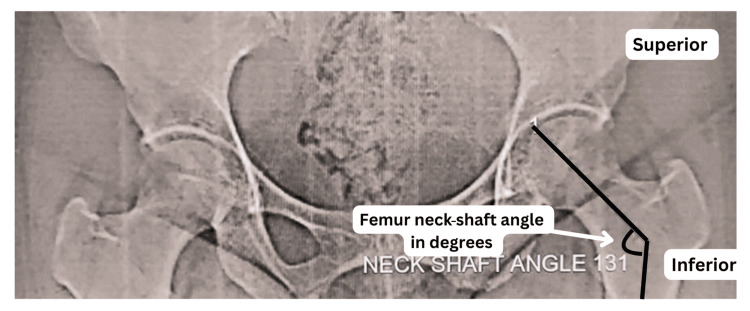
Radiological measurement of the left femoral neck-shaft angle

**Figure 3:**
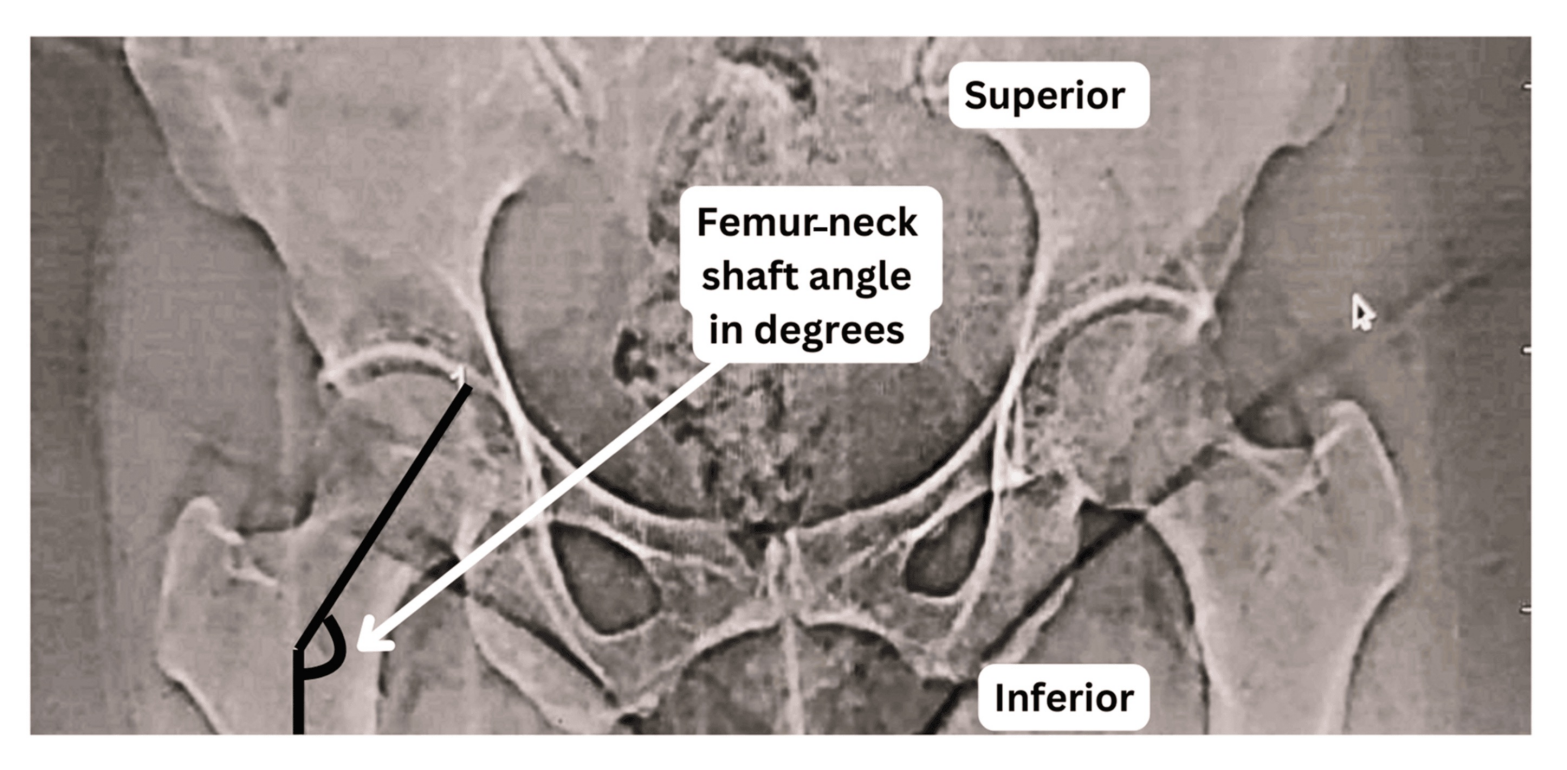
Radiological measurement of the right femoral neck-shaft angle

Radiological analysis was performed using digital X-ray images processed through image analysis software. The femoral NSA was determined by drawing a line along the axis of the femoral neck and another along the axis of the femoral shaft, with the angle formed at their intersection recorded as the femoral NSA (Figures 2, 3). Similar to the osteological assessment, measurements were conducted by two independent radiologists, and the mean of their readings was considered for statistical analysis.

Statistical analysis

Data analysis was performed using IBM SPSS Statistics software, version 26 (IBM Corp., Armonk, NY). Descriptive statistics (mean ± standard deviation) were calculated for osteological and radiological measurements. An independent t-test was applied to compare the femoral NSA values between the right and left NSA, while one-way ANOVA was used to analyze age-related variations in NSA. Pearson correlation analysis assessed the relationship between osteological and radiological measurements with correlation (r): 1.0. A p-value of <0.05 was considered statistically significant for all statistical tests and orthopedic assessments.

Ethical considerations

Institutional ethical approval was obtained from Guntur Medical College, Guntur (approval number: GMC/IEC/037/2024), dated August 22, 2024, before initiating data collection to ensure compliance with ethical research guidelines. Sampling was initiated after approval from the institutional ethical committee. The study was conducted in accordance with the ethical principles outlined in the Declaration of Helsinki (as revised in 2013), which governs research involving human subjects.

## Results

Osteological measurements

One hundred and thirteen dry femur bones (54 left and 59 right) were analyzed to measure the femoral NSA using a goniometer. The mean femoral NSA for the left femur was 116.54° ± 7.81°, while for the right femur, it was 119.93° ± 3.85° (Table 1). The difference between the right and left femoral NSA was statistically significant (p = 0.0047), with the right femur exhibiting a higher mean angle. The observed range of values for both sides varied between 110° and 128°, with the majority of angles clustering between 115° and 125°.

**Table 1:** Femoral NSA in osteological measurements of dry femur bones (mean ± SD) NSA: neck-shaft angle; *significant; p <0.05

Femur side	Mean angle (°)	Standard deviation (°)	p-value (left vs. right)
Left	116.54	7.81	0.0047^*^
Right	119.93	3.85	

Radiological measurements

A total of 30 radiographic images were analyzed, including male and female subjects aged 20 to 80 years. The mean femoral NSA for the right femur was 137.5° ± 5.72°, whereas for the left femur, it was 141.15° ± 6.97° (Table 2). Notably, the left femoral NSA was consistently higher than the right, aligning with the osteological findings.

**Table 2:** Femoral NSA in radiological measurements (mean ± SD) NSA: neck-shaft angle; *significant; p <0.05

Femur side	Mean angle (°)	Standard deviation (°)	p-value (left vs. right)
Left	141.15	6.97	0.0307^*^
Right	137.50	5.72	

Gender-based analysis

In radiological measurements, the mean femoral NSAs for male and female subjects were analyzed (Table 3). Among males, the right femoral NSA was 138.0°, while the left femoral NSA measured 139.8°. For females, the right femoral NSA was 137.33°, and the left femoral NSA was 141.6°. Overall, females exhibited a higher femoral NSA than males, with a more pronounced difference in the left femur.

**Table 3:** Gender-based femoral NSA in radiological measurements NSA: neck-shaft angle; *significant; p <0.05; ^#^non-significant

Gender	Right femur angle (°)	Left femur angle (°)	p-value (right vs. left)
Male	138.0	139.8	0.376^#^
Female	137.33	141.6	0.0385^*^

Age-based analysis

In radiological measurements (total n=30; irrespective of gender), the mean values of the femoral NSA across different age groups (20 to 40 years (n=12), 41 to 60 years (n=9), and 61 to 80 years(n=9)) were analysed and are presented in Table 4. In the 20- to 40-year age group, the right femur angle measured 135.63°, while the left femur angle measured 144.0°. In the 41- to 60-year age group, the right femur angle measured 132.57°, while the left femur angle measured 139.57°. In the 61- to 80-year age group, the right femur angle measured 134.00°, and the left femur angle measured 144.90°.

**Table 4:** Age-based femoral NSA in radiological measurements NSA: neck-shaft angle; *significant; p <0.05

Age group, in years (sample size)	Right femur angle (°)	Left femur angle (°)	p-value (right vs. left)
20-40 (12)	135.63°	144.0°	0.039^*^
41-60 (9)	132.57°	139.57	0.048^*^
61-80 (9)	134.00°	144.90°	0.041^*^

Comparative analysis of osteological and radiological data

Radiological values were consistently higher than osteological values, likely due to soft tissue inclusion and differences in measurement techniques. Despite some variations, a strong correlation was observed between osteological and radiological measurements, confirming the reliability of both methods. The right-sided femoral NSA was lower than the left in radiological measurements, which contrasts with osteological findings where the right side had a slightly higher mean value.

Statistical significance

The difference between left and right femoral NSAs was statistically significant (p <0.05). T-tests and ANOVA analyses confirmed significant variations based on gender and age (p <0.05). Pearson correlation analysis demonstrated a strong positive correlation between osteological and radiological measurements (p <0.05), supporting the accuracy and consistency of the data.

## Discussion

The present study aimed to evaluate the femoral NSA in the adult population. The findings provide significant insights into side-specific (among osteological and radiological measurements), gender-based (radiological measurement), and age-related (radiological measurement) variations in femoral NSA.

Comparison of osteological and radiological measurements

The results demonstrated a statistically significant difference (p < 0.05) between the right and left femoral neck-shaft angles in both osteological and radiological assessments. The osteological measurements revealed a mean NSA of 116.54° ± 7.81° (left) and 119.93° ± 3.85° (right), whereas radiological measurements yielded 141.15° ± 6.97° (left) and 137.5° ± 5.72° (right). These findings indicate that radiological values are consistently higher than osteological values, which can be attributed to soft tissue influence, radiographic projection errors, and methodological differences in measurement techniques. Similar discrepancies between dry bone and radiographic measurements have been reported in previous studies [12, 13], reinforcing the need to consider methodological variations when interpreting femoral NSA values in clinical and forensic applications.

Side-specific variations

A notable finding of this study was the higher femoral NSA in the right femur compared to the left in osteological measurements, whereas, in radiological assessments, the left femur exhibited a greater femoral NSA than the right. These findings may be influenced by asymmetrical loading patterns, biomechanical adaptations related to handedness, and genetic predispositions. While some previous studies have reported no significant side differences [14], others have found similar variations [15], suggesting that population-based factors may contribute to these discrepancies.

Gender-based differences

In radiological measurements, gender-specific analysis revealed that females exhibited a significantly higher femoral NSA than males. The mean femoral NSA for males was 138.0° (right) and 139.8° (left), whereas for females, it was 137.33° (right) and 141.6° (left). These findings align with existing literature, which suggests that females generally have a greater femoral NSA due to anatomical differences, including a wider pelvis and increased femoral anteversion [14]. This anatomical distinction may be necessary in hip biomechanics, susceptibility to joint degeneration, and orthopedic surgical planning. The observed gender-based differences emphasize the need for sex-specific reference values in orthopedic and forensic applications and align with previous studies [13].

Age-related trends in femoral NSA

In radiological measurements, age-wise analysis indicated a gradual decrease in femoral NSA from early adulthood to middle age (41 to 60 years), followed by a slight increase in older individuals (above 60 years). The mean femoral NSA was 135.63° (right) and 144.0° (left) in the 20- to 40-year age group (n=12), 132.57° (right) and 139.57° (left) in the 41- to 60-year age group (n=9), and slightly increasing to 134.00° (right) and 144.90° (left) in the 61- to 80-year age group (n=9). This pattern suggests that age-related bone remodeling, osteoporosis, and adaptive changes in weight-bearing mechanics contribute to femoral NSA variations over time. The slight increase in NSA in older individuals may be associated with compensatory changes due to degenerative alterations in the hip joint [16]. However, definite conclusions may be obtained with a large sample size.

Clinical and orthopedic implications

Higher femoral NSA in females may contribute to their increased susceptibility to hip fractures and osteoporotic changes, emphasizing the importance of early screening and preventive measures. Additionally, the age-related decline in femoral NSA supports the need for bone-strengthening interventions and fall-prevention strategies in aging populations [15].

The findings of this study may be helpful for prosthetic design and orthopedic surgeries, such as total hip arthroplasty (THA) and femoral osteotomy. Population-specific femoral NSA reference values are critical for optimizing implant positioning, reducing postoperative complications, and improving functional outcomes. The observed differences between osteological and radiological femoral NSA values suggest that preoperative planning should account for radiographic distortions and measurement inconsistencies [17].

Limitations of the study

This study, while contributing valuable baseline data on femoral NSA in the adult population of Andhra Pradesh, is subject to certain limitations. The relatively small radiological sample size limits the statistical power and generalizability of subgroup analyses. Additionally, the absence of demographic and clinical data for osteological specimens, such as age, sex, body mass index, and medical history, restricts the ability to assess potential confounding variables. Methodological differences between osteological and radiological measurements, particularly the influence of soft tissue and patient positioning in radiographs, may introduce measurement bias. Furthermore, the study’s cross-sectional design precludes longitudinal assessment of age-related changes in NSA. The lack of functional correlation with clinical or biomechanical outcomes also limits the translational applicability of the findings. Future research should aim to address these limitations through larger, demographically stratified cohorts and integrated clinical, functional, and imaging data.

## Conclusions

This study provides valuable baseline data on femoral NSA in an adult sample in the population of Andhra Pradesh, with significant variations observed based on side, gender, and age. A positive correlation between osteological and radiological measurements supports the reliability of both methods. These findings may be crucial for enhancing orthopedic surgical outcomes and prosthetic designs tailored to regional and demographic characteristics. Future studies with larger sample sizes, stratified by gender and age, should include clinical and lifestyle parameters to further refine these findings and enhance their translational relevance in orthopedic practice and biomedical research.
